# *Pinna nobilis*, the Vanishing Giant: A Comprehensive Review on the Decline of a Mediterranean Icon

**DOI:** 10.3390/ani15142044

**Published:** 2025-07-11

**Authors:** Ilenia Azzena, Chiara Locci, Noemi Pascale, Ilaria Deplano, Riccardo Senigaglia, Fabio Scarpa, Marco Casu, Daria Sanna

**Affiliations:** 1 Department of Veterinary Medicine, University of Sassari, Via Vienna 2, 07100 Sassari, Italy; iazzena@uniss.it (I.A.); c.locci3@phd.uniss.it (C.L.); npascale@uniss.it (N.P.); rsenigaglia@uniss.it (R.S.); marcasu@uniss.it (M.C.); 2 Department of Biomedical Sciences, University of Sassari, Viale San Pietro 43b, 07100 Sassari, Italy; i.deplano@phd.uniss.it (I.D.); fscarpa@uniss.it (F.S.); 3 Department of Chemical, Physical, Mathematical, and Natural Sciences, University of Sassari, Via Vienna 2, 07100 Sassari, Italy

**Keywords:** bivalve mollusc, endangered species, disease, recovery, conservation, refugia, *Haplosporidium pinnae*, review

## Abstract

This review addresses the critical conservation challenges faced by *Pinna nobilis*, the noble pen shell, a keystone umbrella species in Mediterranean marine ecosystems. Since 2016, the species has experienced catastrophic population declines due to mass mortality events likely driven by protozoan, bacterial, and viral infections. Despite these severe circumstances, small resilient populations persist in select estuaries and coastal lagoons across the Mediterranean, offering potential for recovery. We provide a comprehensive overview on research dedicated to *Pinna nobilis*’ biology, genetic variation, disease dynamics, and environmental factors influencing its survival, with a focus on refugia where populations still endure. Remarkably, recent studies have revealed signs of resistance in certain individuals and the potential for hybridisation with *Pinna rudis*. In this context, the possible impact of the increasing occurrence of hybridisation between *Pinna nobilis* and *Pinna rudis* on the conservation of their genetic diversity should be carefully considered. This review highlights the importance of ongoing conservation efforts including habitat restoration, protection of remaining populations, assessment of past and present genetic variability, and the development of captive breeding programmes. We aim to elucidate the need for continued studies on *Pinna nobilis*’ biodiversity, particularly its evolutionary dynamics, genetic makeup, and the interplay of environmental variables influencing its survival and persistence.

## Simple Summary

The noble pen shell, once common in the Mediterranean, is now on the edge of extinction due to a deadly disease. But tiny surviving groups offer hope—some may even be naturally resistant or able to hybridise with relatives for survival. Scientists are racing to rescue and protect this iconic species before it is too late.

## 1. Introduction

Understanding the importance for the conservation of *Pinna nobilis* is crucial given its significant ecological role and critically endangered status. As one of the largest bivalves in the Mediterranean, *Pinna nobilis* contributes to ecosystem balance by filtering water and providing shelter for various marine species [1,2]. However, its population has declined rapidly in recent years due to habitat destruction, pollution, climate change, and diseases caused by various harmful microorganisms, including protozoa, bacteria, and viruses [3,4,5,6,7]. As a consequence, the IUCN has classified it as critically endangered [8]. A thorough analysis of current research is essential to summarise scientific findings, identify gaps in knowledge, and guide future studies. Additionally, reviews play a vital role in raising awareness among environmentalists, policymakers, and the public about the urgent need for protection. They also facilitate the exchange of information among scientists and environmental organisations, encouraging collaboration in conservation efforts. Since *Pinna nobilis* is now at risk of extinction, analysing its current status, threats, and potential recovery strategies is essential for its survival and for maintaining the health of Mediterranean marine ecosystems.

## 2. Materials and Methods

### 2.1. Literature Search

A comprehensive literature review was carried out to assess the conservation status, ecological importance, genetic diversity, and disease-related challenges affecting *Pinna nobilis*, the noble pen shell. To ensure thorough coverage of relevant studies, various academic databases were searched, including Google Scholar, ScienceDirect, Scopus, PubMed, and Web of Science. The primary goal was to identify peer-reviewed research articles containing original data on the biology, population declines, mass mortality events (MMEs), genetics, and conservation measures related to *Pinna nobilis*. Additionally, studies on potential hybridisation with *Pinna rudis* and reports of individuals exhibiting natural resistance were analysed to explore future conservation possibilities.

### 2.2. Search Strategy

The search process involved specific keywords and phrases related to *Pinna nobilis*. Key search terms included “*Pinna nobilis* mass mortality events,” “*Pinna nobilis* conservation strategies,” “*Pinna nobilis* genetic diversity,” “*Pinna nobilis* habitat restoration,” “*Pinna nobilis* environmental factors,” “*Pinna nobilis* population resilience,” and “*Pinna nobilis* hybridisation with *Pinna rudis*.” Search queries were refined using Boolean operators (AND/OR) to enhance the accuracy and relevance of retrieved studies. To maintain a broad and well-rounded review, studies from all publication years were considered, incorporating both foundational research and the latest findings. The reference lists of selected articles were systematically examined to discover additional relevant sources, which were subsequently obtained from the specified databases. To maintain scientific integrity, non-academic sources, such as commercial websites or unverified reports, were excluded.

## 3. *Pinna nobilis* (Linnaeus 1758): Overview and Ecological Role

Comprehending the biology and ecological function of *Pinna nobilis* is crucial for the successful conservation and management of Mediterranean marine ecosystems. By studying its habitat preferences, feeding behaviour, reproductive patterns, and interactions with other species, we can better assess the threats it faces and develop targeted protection strategies.

In this context, *Pinna nobilis*, commonly known as the fan mussel or noble pen shell, is a long-lived bivalve endemic to the Mediterranean Sea, belonging to the family Pinnidae (Mollusca: Bivalvia) [9,10]. Its taxonomy has undergone revisions and currently includes three genera: *Pinna*, *Atrina*, and *Streptopinna* [11].

This species inhabits coastal waters at depths ranging from 0.5 to 60 m, preferring soft-bottom environments, particularly mixed seagrass meadows [12].

**Figure 1:**
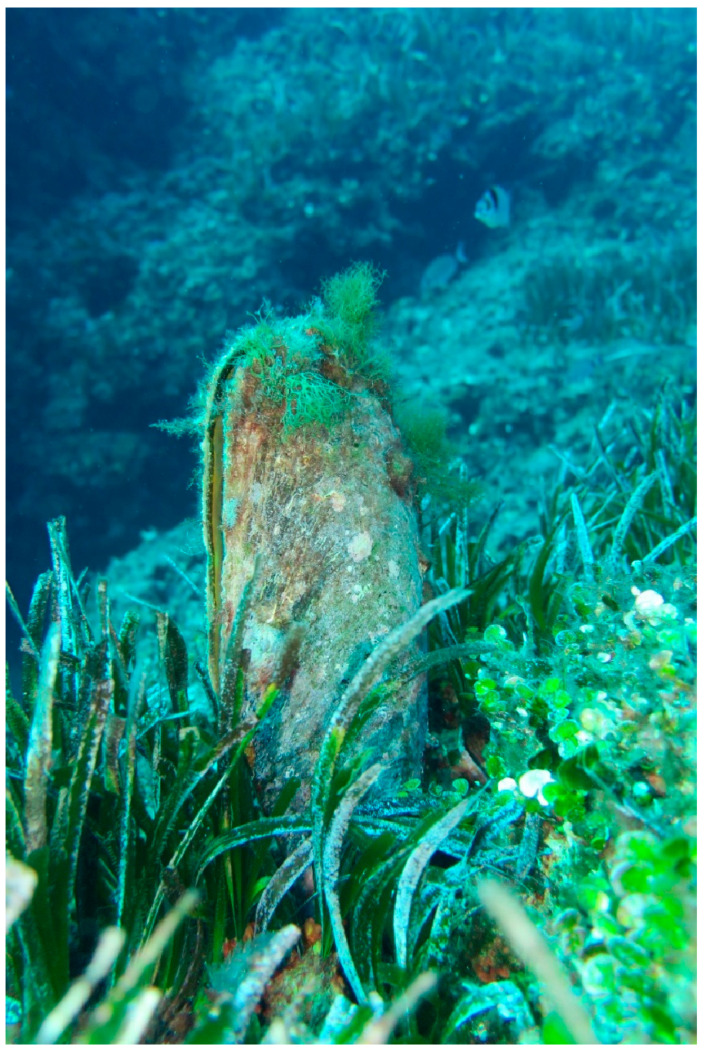
*Pinna nobilis* in Costa Paradiso (Northwest Sardinia) before MMEs. The image depicts an underwater scene featuring a fan mussel (*Pinna nobilis*) partially buried in a seagrass meadow of *Posidonia oceanica*. The mussel’s shell is covered with algae and other marine organisms, highlighting its integration into the ecosystem. Photo credits: Dr. Elisabetta Lutzu.

As a filter feeder, *Pinna nobilis* thrives in *Posidonia oceanica* meadows, playing a crucial role in the ecosystem [1]. While most individuals are found in seagrass habitats, some have been observed on bare sandy bottoms or maërl beds (shallow, hard substrates formed by calcareous red algae) [3,12,13,14,15,16] (see Figure 1).

To anchor itself, *Pinna nobilis* produces byssus threads from a specialised gland in its foot, attaching firmly to sand, pebbles, and seagrass roots [1]. Each individual generates between 20,000 and 30,000 filaments, which require continuous reinforcement to maintain stability [1,16,17]. This attachment method leads to an anisomyarian condition, where the anterior adductor muscle is significantly reduced compared to the posterior muscle—an adaptation that enhances stability in dynamic underwater environments [18].

The shell of *Pinna nobilis* is fan-shaped, thin, and fragile, reaching up to one metre in length [16,19]. Juveniles display external spines, which erode over time [1]. Internally, the anterior section of the shell is lined with a smooth, iridescent nacre layer. Shell morphology exhibits minor regional variations influenced by environmental factors such as hydrodynamics and substrate type [16,20].

Gaping activity, essential for feeding, respiration, and metabolism, follows a diurnal pattern [21]. The shell typically opens during the day and closes at night, with exceptions under moonlight or during storms. Population-wide synchronisation of gaping behaviour suggests a uniform response to environmental stimuli [21].

*Pinna nobilis* is considered a long-lived mollusc, with individuals that can reach up to 45-50 years old, as reported in Port-Cros National Park (Provence, France) [22]. Nonetheless, our knowledge concerning the life expectancy of *Pinna nobilis* is still limited due to the challenging nature of developing an experimental approach that enables the evaluation of the species’ maximum lifespan in undisturbed environments [22].

As a filter feeder organism, *Pinna nobilis’* gills are composed of outer and inner layers that move both upwards and downwards. These filaments are intricately interconnected, forming a densely compacted gill structure that creates a cavity leading into a spacious square chamber and ultimately into the upper mantle cavity. Notably, the outer gill filaments are rich in glandular cells producing mucus, which covers the gill and forms a net for particle capture and filtration [23].

Studies on its diet reveal that *Pinna nobilis* predominantly consumes detritus (95% of its intake), along with phytoplankton, micro- and mesozooplankton, and pollen grains [24]. The diet can vary by location, with phytoplankton being a primary food source in some regions [16,25]. An analysis of fatty acids in different tissues suggests that smaller individuals rely more on detritus, while larger ones consume more polyunsaturated fatty acids, indicating a shift in diet as they grow [2,26].

Reproduction in *Pinna nobilis* is characterised by its sequential hermaphroditism, with asynchronous maturation to avoid self-fertilisation [27]. The reproductive cycle has four main phases, influenced by environmental and internal factors, with sexual maturity reached at two years [27,28,29]. The duration of the larval stage of *Pinna nobilis* is not yet confirmed; however, it is generally considered to be less than 10 days [12,30,31]. Nevertheless, based on data from other species within the family Pinnidae, it might extend to several weeks [32,33,34].

Recruitment of *Pinna nobilis* is influenced by several factors, including rising seawater temperatures, which can reduce reproductive output and alter spawning times [35]. Indeed, elevated temperatures can influence spawning periods and shorten the planktonic larval stage, promoting earlier settlement near the parental area [31]. In contrast, lower temperatures extend the dispersal phase, allowing larvae to reach more distant regions and fostering connectivity among *Pinna nobilis* populations [31]. However, limited studies have modelled the recruitment of *Pinna nobilis*, highlighting the need for more research [36].

### Ecological Role

**Figure 2:**
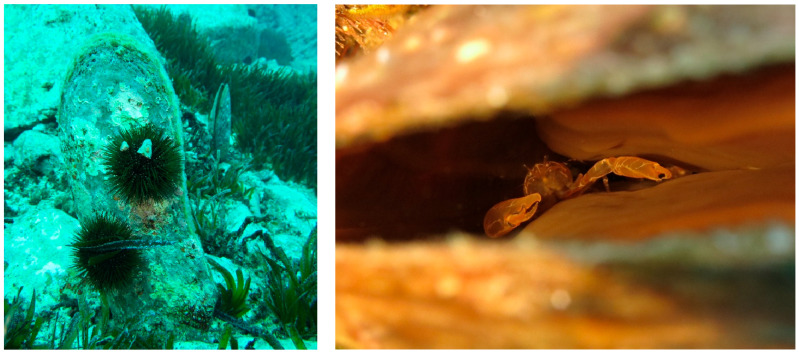
*Pinna nobilis* and its associated fauna in Costa Paradiso (Northwest Sardinia) before MMEs. On the left in the figure, a fan mussel (*Pinna nobilis*) with sea urchins attached to its shell, likely feed on encrusting organisms. On the right in the figure is a close-up of the mussel’s interior, revealing a shrimp (*Pontonia pinnophylax*) residing within. Photo credits: Dr. Elisabetta Lutzu.

*Pinna nobilis* plays a crucial role in marine ecosystems beyond its function as a filter feeder. By enhancing water clarity and contributing to overall environmental health [2], it helps maintain ecosystem stability. Additionally, its hard shell serves as a substrate for various benthic species, thereby promoting biodiversity [16] (see Figure 1 and Figure 2).

This ecological role extends to supporting a diverse epibiontic community, including molluscs, annelids, crustaceans, and sponges, which further enrich local biodiversity [37,38,39,40]. Notably, the shrimp *Pontonia pinnophylax* and the crab *Nepinnotheres pinnotheres* live in association with *Pinna nobilis*, though their interactions remain poorly understood [41,42]. By creating habitats in soft-bottom environments, *Pinna nobilis* significantly enhances overall biodiversity [16].

## 4. Exploitation, Decline, and Conservation of *Pinna nobilis* in the Mediterranean

Acknowledging the history of *Pinna nobilis* exploitation is vital for effective conservation and management. By recognising the long-term pressures, such as overharvesting, that have contributed to the species’ decline, we can better identify the continuing threats to its survival. This knowledge facilitates the development of more targeted protection strategies, addressing both past and present challenges. It also emphasises the importance of enforcing conservation measures to prevent further damage, supporting more successful recovery efforts for vulnerable species.

Over the centuries, *Pinna nobilis* has faced various forms of exploitation, which have significantly impacted its populations. In the past, the inner layer of shells was used for ornamental and decorative purposes and valued for its aesthetic qualities in crafting jewellery, buttons, and other decorative items [3]. Additionally, the byssus—the fine threads produced by *Pinna nobilis* to anchor itself to the seabed—was highly valued for making the so-called “sea silk”, a rare and luxurious fabric. This demand had a major role in the historical overexploitation of the species, particularly in Southern Italy, where regions such as Apulia (with the area of Taranto) and Sardinia (with the island of Sant’Antioco) had a longstanding tradition of harvesting byssus to produce fine textiles [3]. This practice dates back to ancient times, with records of its existence found as early as the Greek and Roman periods [43,44].

Moreover, the distinct shape of its shell made it desirable for collectors, contributing to their overharvesting in some areas [12,45]. Furthermore, around the middle of the twentieth century, a new and widely adopted practice began: the abductor muscle of *Pinna nobilis* started being used in cooking, while the rest of the organism was utilised as fishing bait [43,46].

Apart from these, the populations of *Pinna nobilis* were also severely impacted by indirect human activities, including boat anchoring, pollution, and habitat fragmentation [47,48]. Therefore, the populations of *Pinna nobilis* throughout the Mediterranean Sea started a demographic decline that escalated significantly by the late 1980s [49].

### Protection Regime and Its Effects over Time

To invert the trend of demographic decline, *Pinna nobilis* was included at the beginning of the 1990s in a full protection regime under the Annex IV of the EU Habitats Directive (European Council Directive 92/43/EEC) and Annex II of Barcelona Convention (SPA/BD Protocol 1995). Moreover, many countries have enacted their own legislative measures, establishing conservation protocols to address the historical exploitation and continuous threats facing *Pinna nobilis*. In Italy, within the context of the Marine Strategy Monitoring Program, the noble pen shell was identified as a species of particular interest among the Mediterranean species worthy of attention, as outlined in Article 11 of Legislative Decree 190/2010 [43].

Despite the great commitment of the European community and individual nations in the protection of this species, some areas of the southwestern Mediterranean Sea manifest poor adherence to these conservation policies and some individuals continue to be illegally harvested for consumption [50] or artisanal purposes [8].

Nevertheless, this pattern of illegal actions does not represent the prevailing trend among the nations surrounding the Mediterranean Sea.

## 5. Mass Mortality Events in *Pinna nobilis*: Causes, Spread, and Scientific Investigations

Mass mortality events (MMEs) affecting *Pinna nobilis* have raised significant concern across the Mediterranean. Although the specific cause remains unclear, current research suggests that these events are the result of a multifactorial disease processes involving both biotic agents—such as *Haplosporidium pinnae*, mycobacteria, and viruses—and abiotic stressors, including elevated sea temperatures and environmental changes [43,51,52]. These factors likely act synergistically, compromising the host’s immune response and leading to increased mortality. In the following sections, we present a point-by-point overview of the key findings to date regarding each of the potential contributing factors.

### 5.1. Timeline of MME Occurrences

After years of stability under protection measures, an alarming event occurred in 2016. *Pinna nobilis* populations along the central and southern coasts of Spain, including the Balearic Islands, began to experience MMEs, with mortality rates reaching up to 100% [4]. Histological analysis of the affected individuals revealed the presence of a haplosporidian-like parasite in their digestive glands [4,53].

Since the earliest outbreak, numerous MMEs have been reported across the Mediterranean, initially spreading from Spain to France and Italy, and later extending to Croatia, Bosnia and Herzegovina, Greece, and Turkey [4,5,54,55,56,57,58,59,60].

**Figure 3:**
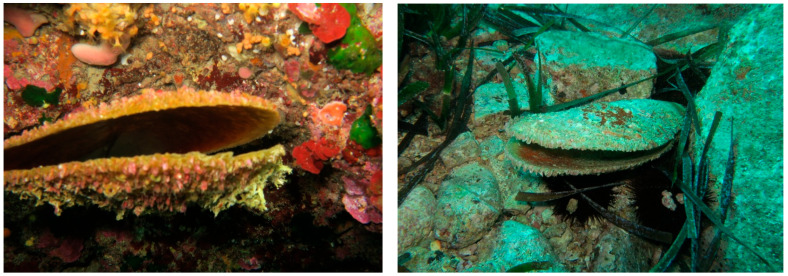
*Pinna nobilis* in Costa Paradiso (Northwest Sardinia) after MMEs in 2018. The figure depicts dead individuals scattered among rocks and seagrass (on the **left**), and with sea urchins (on the **right**), likely feeding on decomposing tissue and epibionts. Photo credit: Dr. Elisabetta Lutzu.

In the early phase of the outbreak, affected individuals exhibited a delayed shell-gaping response to external stimuli, clearly indicating compromised health. Some individuals remained upright but displayed collapsed tissue at the bottom of their shells, while others had already died, leaving empty shells that were often colonised by other marine species, as shown in Figure 3 [4,43]. In cases where the individuals remained alive but weakened, their delayed reactions made them highly vulnerable to predation.

### 5.2. Identifying the Causes of Mass Mortality Events

Following the first reports of MMEs [4], researchers launched multiple studies to identify the pathogens responsible. Protozoan parasites—specifically haplosporidian endoparasites—were among the first suspected causes, as they have been linked to large-scale die-offs in various bivalve populations [4,5,53,54,55,61,62].

Haplosporidian parasites have a complex life cycle consisting of two main stages:

- Plasmodium Stage—A single or multinucleate phase present in the host’s tissues.
- Sporulation Phase—The production of resilient spores, which are released into the environment upon the host’s death [51,53].

The first detailed description of the haplosporidian-like parasite in *Pinna nobilis* reported systemic infections affecting connective tissues and causing damage to the digestive tubules [51]. Based on these findings, subsequent research identified the parasite as *Haplosporidium pinnae*, initially considering it to be exclusive to *Pinna nobilis* [5].

Throughout the infection process, the pathogen develops into the plasmodium stage, during which it is typically found within the haemocytes and connective tissues of the mantle and digestive gland of *Pinna nobilis*. Sporulation then occurs primarily in the digestive tubules and occasionally in the gut epithelium [51].

However, further investigations revealed a more complex picture because *Haplosporidium pinnae* was detected in other bivalve species, such as *Mytilus galloprovincialis* and *Ruditapes decussatus*, sampled a few years prior to the 2016 outbreak of MMEs [43]. This finding challenges the earlier suggestion [5] that the parasite could be species-specific to *Pinna nobilis*. Instead, it indicates that *Haplosporidium pinnae* is not exclusive to *P. nobilis* and may have been present, in several Mollusca species, in the Mediterranean prior to the 2016 MME.

As investigations into the causes of MMEs continued, researchers identified additional pathogens that may have a significant role in the decline of *Pinna nobilis* populations. Among these, bacterial species, particularly *Mycobacterium* spp. and *Vibrio* spp., were frequently detected in *Pinna nobilis* tissues [6,43,57,58,60,62,63,64]. Notably, three new taxonomic groups of *Mycobacterium* spp. were identified in diseased specimens, together with other bacteria such as *Rhodococcus erythropolis* and *Perkinsus* sp., which were often found in association with *Mycobacterium* [43,63].

More recently, a virus from the order *Picornavirales*, classified within the genus *Sogarnavirus* and replicating inside immune cells, has been detected in some *Pinna nobilis* specimens [7,65]. The presence of this virus appears to compromise the host’s immune function, as evidenced by a decline in haemocyte levels, which likely increases vulnerability to opportunistic infections [7]. These findings indicate that the virus weakens the immune system and may become particularly harmful under stressful conditions such as those experienced in captivity [65]. Nonetheless, additional research is necessary to verify the virus’s identity and clarify its pathogenic impact.

Remarkably, not all individuals infected with bacteria or viruses were found to carry *Haplosporidium pinnae* [43,52,66].

The discovery of multiple pathogens and their interactions suggests that the MMEs result from a complex, multifactorial disease rather than a single causative agent [43].

Understanding the full scope of this crisis remains an ongoing challenge, requiring continued research and conservation efforts to prevent the extinction of *Pinna nobilis* in the Mediterranean.

## 6. Genetic Structure and Variability of *Pinna nobilis*: Insights Before and After Mass Mortality Events

Understanding the genetic diversity of *Pinna nobilis* is essential for guiding conservation and recovery efforts, especially considering the MMEs. Genetic diversity has a key role in the capacity of a species to adapt to environmental shifts and withstand threats and stressors such as diseases and climate change. Encouragingly, a recent study [67] analysing Spanish populations of *Pinna nobilis* found that despite severe declines, the resilient Mar Menor population retains high genetic diversity and unique traits, further boosting hope for the survival of the species. In fact, without adequate genetic variation, populations become increasingly vulnerable to extinction [68,69,70].

This section explores how the genetic structure of *Pinna nobilis* has been shaped over time, focusing on studies conducted before, during, and after the onset of MMEs [71,72,73,74,75,76,77,78,79,80].

### 6.1. Before Mass Mortality Events

Before 2016, relatively few studies focused on the genetic diversity of *Pinna nobilis*, primarily assessing how past conservation efforts impacted the species. One of the earliest investigations [71] analysed four populations in the Aegean Sea using mitochondrial markers (COI gene and 16S rRNA). The study revealed notable haplotypic diversity in the Cytochrome c Oxidase subunit I gene (COI), although genetic differentiation among populations was minimal, indicating a lack of genetic structuring within the region.

Subsequent research on the Tunisian coastline [72] observed a decreasing gradient of genetic variability from the northern to eastern coast, likely influenced by variations in the hydrodynamic conditions of the studied areas.

In 2013, a comprehensive Mediterranean-scale assessment of the genetic variability of *Pinna nobilis* was conducted, covering 25 sites across the Mediterranean Sea [73]. To broaden the geographic coverage of earlier research, the authors employed the same mitochondrial markers (COI gene and 16S rRNA), incorporating their newly generated sequences with the previously published data [71,72]. The study revealed considerable genetic variability within three major marine ecoregions—the western Mediterranean Sea and the Ionian Sea, the Adriatic Sea, and the Aegean Sea including the Tunisian coast—and distinguished two main genetic clusters, clearly separating the eastern and western Mediterranean populations. Notably, the study proposed that populations in the Venetian lagoon might represent a distinct genetic group warranting separate conservation efforts.

Subsequent years witnessed the advent of microsatellite markers, which emerged as instrumental tools for the investigation of genetic diversity and connectivity [74]. These markers were subsequently applied in a study of populations sampled between 2010 and 2011 along the Spanish Mediterranean coast [75]. Using a multidisciplinary approach that combined population genetics with hydrodynamic modelling, the study revealed high genetic diversity within populations and significant genetic differentiation among post-larvae, though not among adult individuals.

A subsequent study [76] expanded this work across the western Mediterranean (French and Spanish coastlines), integrating microsatellites and mitochondrial COI sequences. The findings confirmed high genetic diversity and limited inter-population differentiation in *Pinna nobilis*. Furthermore, the results supported the existence of a single lineage that likely underwent a recent expansion from a small ancestral population [76], consistent with the hypothesis proposed in a previous study [73], which suggested an eastward expansion across the Mediterranean—likely driven by marine currents such as the Algerian Current—and followed by one or more founder events that led to reduced genetic diversity and the presence of private haplotypes in the Aegean and Tunisian populations.

In this context, a recent study [77] aimed to clarify the evolutionary history of *Pinna nobilis*, including its phylogenetic relationships within the Pinnidae family—particularly with its close relative *Pinna rudis*—and to explore evolutionary models by tracing its divergence in the Mediterranean Sea, leading to its current critically endangered status. The study analysed approximately 500 mitochondrial COI gene sequences from *Pinna nobilis* populations across the entire Mediterranean region, revealing that the species diverged from an Atlantic ancestor around 2.5 million years ago. This Atlantic ancestor likely entered the Mediterranean Sea during the Zanclean flood 5.33 million years ago, a period when the Mediterranean was reconnected to the Atlantic Ocean, enabling the first colonisation of the central western Mediterranean seafloor. Following its emergence, early *Pinna nobilis* populations spread into the Adriatic Sea and then dispersed into the eastern Mediterranean Sea. Moreover, the high number of sequences from the Adriatic Sea—an area previously understudied—provided evidence of genetic interconnection between Adriatic and western populations. Finally, the study also recognised *Pinna rudis* as the sister species of *Pinna nobilis*.

### 6.2. During and After Mass Mortality Events

Everything changed in 2016 with the onset of MMEs. These die-offs raised urgent questions about the genetic resilience of the species, prompting new studies to assess whether surviving populations maintained sufficient genetic diversity.

One of the largest post-MME studies [78] analysed 960 individuals along the Gulf of Lion in France. Despite the severe population decline, genetic diversity remained high, and no significant differentiation was found among surviving populations. This suggested that, at least in this region, *Pinna nobilis* remained genetically uniform and interconnected.

In a follow-up study [79], *Pinna nobilis* populations in Peyrefite Bay (France) were monitored over several years. While genetic diversity remained consistently high, significant differences emerged over time. The study also revealed a striking pattern of family clustering, with one dominant family thriving—likely due to more successful spawning or higher larval survival. Although many individuals were closely related, there was no evidence of self-recruitment.

Elsewhere, researchers have investigated genetic patterns in the eastern Mediterranean, focusing on Greek and nearby waters [80], including Karpathos, Lesvos, Crete, Chalkidiki, Attica, and the Amvrakikos Gulf. The results showed genetic connectivity within this region, with no significant differentiation among populations. However, a clear genetic distinction between the western and eastern Mediterranean was confirmed, likely driven by oceanographic barriers.

Finally, a recent study [67] comparing two Spanish populations—one surviving and one extinct—found that, despite severe declines caused by MMEs, the surviving *Pinna nobilis* population in the Mar Menor lagoon has retained high levels of genetic diversity and unique alleles compared to the extinct populations of the Cabrera National Park. This suggests that, although the population experienced a dramatic reduction in size, there is no evidence of a recent genetic bottleneck, and the group appears to be near Hardy–Weinberg equilibrium. Overall, the Mar Menor population exhibits levels of genetic diversity comparable to other surviving lagoon populations [78].

These comprehensive genetic studies have led to significant advancements in our understanding of *Pinna nobilis*, encompassing its population structure, population connectivity, and evolutionary history. It has been hypothesised that the high levels of genetic diversity will have an important role in the resilience and survival of the species and may contribute to the recovery of this critically endangered species [68,69,70]. As we move forward, the protection of *Pinna nobilis* is not merely a matter of preserving a species; it is also about maintaining the delicate balance of the Mediterranean ecosystem and ensuring that future generations can witness the presence of this iconic marine giant.

It should be noted that the development of non-lethal sampling techniques has facilitated the acquisition of genetic data without causing harm to the subjects [71,77,80,81]. One method [71] involved the gentle opening of the shell underwater with a wooden stick to collect a small mantle tissue sample using forceps. An alternative approach [80] entailed the utilisation of a diminutive rod inserted between the valves to maintain their slight opening, accompanied by the employment of a soft brush to collect mucus and tissue samples from within the shell.

## 7. The Last Strongholds: Surviving Populations of *Pinna nobilis* in the Mediterranean

This section underscores the critical role of the remaining *Pinna nobilis* populations in isolated refuges across the Mediterranean, highlighting their vulnerability and the possible factors contributing to their survival.

*Pinna nobilis* populations have been severely impacted by MMEs that began in 2016, leaving only a few surviving groups. These remaining populations are primarily found in estuaries and isolated coastal lagoons across France, Italy, and Spain [82], while in the eastern Mediterranean, the Sea of Marmara has also become a vital refuge for the species [83]. Sadly, by 2023, some of these populations have already disappeared.

Notable surviving populations are found in specific areas across the Mediterranean and beyond. In Spain, key populations are located in Fangar Bay, the Ebro Delta, and the Mar Menor [84]. In France, the Rhône Delta and Thau lagoon continue to support *Pinna nobilis* [85], while Corsica offers refuge in the Diana and Urbino lagoons. In Italy, populations can still be found in the Venetian lagoon [86], Faro Lake in Sicily [87,88], and Sant’Antioco Island in Sardinia (authors’ personal observation). The species also persists in the Ionian Sea, particularly in Amvrakikos Gulf [80,89], as well as in the eastern Mediterranean, notably in the Gulf of Kalloni on Lesbos, Greece [89,90], as well as along the southern coast of Marmara Island in Turkey [83]. Additionally, in Algeria, the bays of Arzew and Kristel host *Pinna nobilis*, while in Tunisia, residual populations persist in Bizerte Lagoon, the waters off Monastir, and around the Kerkennah Islands [91]. Despite the challenges, these refuges offer hope for the survival of this species.

The reasons these populations have survived while others have perished are not yet fully understood. One possibility is that differences in salinity and temperature between lagoons and open waters provide a natural barrier against infection and disease [55,82,92]. Some studies suggest that both extremely high and low salinity levels, as well as cooler temperatures, may limit the spread of pathogens affecting *Pinna nobilis* [91]. However, the exact salinity and temperature ranges that offer protection are still unknown.

Despite their role as refuges, these coastal lagoons face significant threats of their own. Pollution, habitat destruction, and overexploitation put these ecosystems at risk [93,94]. Additionally, because lagoons have limited water exchange with the open sea, they are vulnerable to eutrophication, which can lead to harmful algal blooms that negatively affect both marine organisms and human health [95].

## 8. Can *Pinna nobilis* Recover from an Infection Provoked by *Haplosporidium pinnae*? The Case of the Mar Menor Lagoon

The situation of *Pinna nobilis* in the Mar Menor lagoon, Spain, is a key example of how multiple threats can impact the survival of a species. Recent studies have allowed us to explore whether *Pinna nobilis* can recover from the potentially harmful effects of *Haplosporidium pinnae*, one of the etiological agents likely responsible for its decline.

The population of *Pinna nobilis* in the Mar Menor lagoon has been under close monitoring since 2014 [96]. Early surveys showed stable numbers, but densities were lower than expected, with few juveniles and large individuals, likely due to predation by the gastropod *Hexaplex trunculus* and pollution. In 2015, an algal bloom caused by agricultural runoff led to widespread mortality, especially at depths below 2.5 m. While some recovery was observed in 2017, the population faced new challenges, including the following: (i) invasive *Hydroides* polychaetes blocking the shells of *Pinna nobilis* in 2017, impairing their ability to feed; (ii) algal growth on the shells, slowing growth rates; and (iii) excessive rainfall in 2018 and 2019, which worsened water quality, reduced oxygen levels, and caused salinity fluctuations, further stressing the population.

By 2019, *Haplosporidium pinnae*, one of the parasites responsible for the MMEs, was detected in the lagoon. This parasite may have entered the lagoon through extreme weather events, or it might have been present since 2017, lying dormant until conditions allowed it to activate. Interestingly, molecular analysis in June 2020 revealed that three *Pinna nobilis* individuals, previously infected with *Haplosporidium pinnae* in November 2019, had recovered from the infection, suggesting potential resistance [96].

To date, the population of *Pinna nobilis* in Mar Menor is represented by a small number of individuals, highlighting the urgent need for continued monitoring and conservation efforts [84,97]. For successful conservation, it is vital to focus on researching genetic resistance, environmental influences, and habitat protection. Safeguarding these remaining populations is crucial for ensuring the long-term survival of the species in the Mediterranean.

## 9. Hybridisation Between *Pinna nobilis* and *Pinna rudis*: Implications for Adaptation and Disease Resistance

Hybridisation between different species can have a significant role in evolution, offering new ways for a species to adapt to changing environments [98]. It can sometimes blur the boundaries between species [99], and in other cases, create entirely new species with unique traits that might give them a competitive advantage in difficult environments [100]. Hybrids often possess a combination of characteristics from both parent species, which can help them thrive in different ecological conditions [101,102]. This process of interspecific hybridisation is common in many animal groups and is important for their ability to adapt to environmental changes [103,104].

This section explores the significance of hybridisation between *Pinna nobilis* and *Pinna rudis*, highlighting how this process might enhance the adaptability of the species to environmental challenges. Hybridisation between these two species was first documented in Cabrera National Park, Spain, where researchers found several empty shells displaying features of both species [104]. Additionally, three live individuals displayed intermediate traits, such as a mix of shell shape and mantle colour (see [104] for the picture of these hybrids). Genetic testing confirmed that these individuals were hybrids of *Pinna nobilis* and *Pinna rudis*. Interestingly, the hybrids showed variations between individuals, and sometimes it was difficult to distinguish them from the parent species based on morphology alone. In this context, a recent study [105] introduced a molecular multiplex PCR technique that employs species-specific primers targeting the nuclear internal transcribed spacer (ITS) regions of *P. nobilis* and *P. rudis*, permitting precise identification of each species as well as the reliable detection of hybrid individuals.

While limited information is available on the biology and ecology of *Pinna rudis*, the occurrence of hybridisation suggests that the two species may spawn around the same time, likely between May and July, with overlapping periods of gamete release. Cabrera National Park is an interesting study site because it has a relatively high abundance of both species, which is unusual for the Mediterranean.

There remains some uncertainty about the classification of these hybrids. Genetic tests suggest they are likely first-generation hybrids, but other data show differences in their genetic traits, making it hard to fully classify them. Two of the hybrids were found in areas where *Pinna nobilis* usually lives, while the third was found in a habitat typical of *Pinna rudis*. This raises questions about whether the hybrids are pure first-generation or the result of multiple generations of introgression. It is also unclear whether these hybrids are fertile and can produce offspring [104].

Preliminary evidence suggests that hybrids may exhibit resistance to *Haplosporidium pinnae*, possibly because they share immune-related genetic traits with resistant individuals of *Pinna nobilis* and *Pinna rudis* [106]. In particular, resistant individuals carry a specific version of the TLR-7 gene, which is likely a key factor in their disease protection [106]. This implies that hybrids, as for *Pinna rudis,* may possess genetic traits that help resist the disease. However, more research is needed to understand whether these hybrids are capable of reproducing and transmitting this resistance to future generations. In this context, the potential impact that a possible increasing occurrence of hybridisation between *Pinna nobilis* and *Pinna rudis* may have on the conservation of their genetic variability should be evaluated.

## 10. Conservation and Restocking Efforts for *Pinna nobilis* in the Mediterranean

As a results of the MMEs, the conservation status of *Pinna nobilis* has been reassessed and classified as critically endangered [8]. In response to this critical situation, various Mediterranean organisations have launched ex situ conservation efforts, focusing on captive breeding and reintroduction programmes [107,108,109]. Significant emphasis has also been placed on protecting the few remaining wild populations of *Pinna nobilis* in the Mediterranean Sea [107]. To support these efforts, the European Union funded two ongoing LIFE projects focused on the conservation and restocking of *Pinna nobilis*: LIFE PINNARCA and LIFE PINNA. These two conservation programmes are crucial to the species’ survival, offering hope for its recovery and providing valuable insights into long-term conservation strategies.

### 10.1. LIFE PINNARCA

LIFE PINNARCA is a European project aimed at conserving *Pinna nobilis* populations throughout the Mediterranean Sea. Its primary goal is to prevent the species’ extinction through a multifaceted approach that includes raising public awareness, fostering collaboration among stakeholders, collecting data on remaining populations, and implementing active recovery measures. The project spans various Mediterranean regions, involving multiple marine protected areas in Spain and Italy, such as Punta Campanella and Cileno, as well as lagoons and semi-enclosed bays like the Gulf of Kalloni in Greece, Mar Menor in Spain, Brusc Lagoon in France, and the Ebro Bays in Spain. By engaging local communities and scientific researchers, the project aims to monitor and restore habitats essential for the survival of *Pinna nobilis*.

### 10.2. LIFE PINNA

LIFE PINNA focuses on conserving and restocking *Pinna nobilis* populations in the western Mediterranean and Adriatic Sea. The project has two main objectives: protecting and monitoring the remaining individuals in these regions and developing captive breeding methods to reintroduce disease-resistant individuals into suitable habitats. This project involves a collaborative effort across Italy (Sardinia, Liguria, Friuli Venezia Giulia, and Tuscany) and Slovenia, bringing together several organisations.

The first phase of the project involves comprehensive environmental and health assessments conducted across various Mediterranean habitats, focusing on areas in northwestern Liguria, northwestern Sardinia, and the Upper Adriatic. Surviving *Pinna nobilis* individuals undergo genetic analysis to identify lineages associated with disease resistance. In addition, efforts are made to monitor pathogens through bivalve filter sentinel species in these regions to ensure that potential restocking sites are safe for reintroduction. During the second phase, disease-resistant individuals of *Pinna nobilis* sourced from the Upper Adriatic are bred in captivity for reproduction and later transplanted into designated pilot areas, selected based on environmental suitability and molecular analyses. This multi-step approach, combining environmental monitoring, captive breeding, and reintroduction, aims to create a replicable model for the restoration of *Pinna nobilis* in other regions.

## 11. Ex Situ Conservation of *Pinna nobilis*: Transport, Captive Maintenance, and Reproduction Challenges

Conservation efforts have increasingly focused on ex situ approaches, including transport, captive maintenance, and controlled reproduction, as potential strategies for restoring wild populations (e.g., LIFE PINNA, LIFE PINNARCA). Despite significant advancements, several challenges remain, particularly regarding the larval development of *Pinna nobilis* in captivity [110].

Several studies have investigated transport protocols and methods for maintaining *Pinna nobilis* in captivity, often using other Pinnidae species as models to refine techniques [110,111,112,113,114]. These studies highlight various difficulties associated with keeping pinnids in controlled environments, including stress-related responses and acclimatisation challenges. Nonetheless, aquaculture remains a crucial conservation tool, offering a potential means to preserve and repopulate *Pinna nobilis* populations [115,116].

Several transport protocols have been developed and standardised across the Mediterranean [110,112,113,117,118]. These typically involve the use of aerated seawater tanks [110,113] or alternative methods such as coolers and iceboxes [112,118]. While various protective measures such as controlling the number of individuals transported, regulating temperature, and ensuring proper aeration have been implemented to minimise stress, translocation can still induce physiological strain and, in some cases, trigger gamete release [109,110].

After transportation, the individuals were maintained in seawater tanks, where potential pathogen contamination was eliminated through cartridge filtration combined with UV sterilisation. To replicate optimal environmental conditions, key factors such as aeration, photoperiod, water temperature, and food availability were carefully controlled [112,113,114,119].

Despite the success in maintaining *Pinna nobilis* in controlled environments, achieving consistent reproductive success remains a significant challenge. While gamete release and fertilisation have been documented, reports of self-fertilisation [109,110] contrast with earlier findings [27] that described *Pinna nobilis* as a non-sequential hermaphrodite, a reproductive strategy thought to prevent self-fertilisation. This discrepancy raises questions about the reproductive dynamics of the species in artificial conditions and necessitates further investigation.

The development of *Pinna nobilis* larvae in captivity is influenced by several factors, including water temperature, light cycle (photoperiod), and food quantity. Research [120] has suggested that the larvae would require a specific microalgae diet for their growth, consisting of 25 cells per μL of *Chaetoceros calcitrans*, 33.3 cells per μL of *Pavlova lutheri*, and 100 cells per μL of *Isochrysis galbana*, together with an appropriate light cycle. When other combinations of food, temperature, and photoperiod were tested, they do not seem to have produced viable larvae [120]. However, in this study, all larvae died before reaching 110 μm in shell length, suggesting that these conditions, despite being proposed in the original paper, could not lead to successful larval development to the pediveliger stage.

Supporting this finding, another study [110] observed rapid larval development, likely due to the elevated water temperature (~25 °C) during rearing. However, the researchers also noted that, beyond temperature, varying microalgae concentrations may have limited larval development. Specifically, the cell concentration used was 10 times lower than that recommended in the earlier study [120], which likely hindered larval growth [110].

Currently, existing methods only allow *Pinna nobilis* larvae to be reared up to approximately 100 microns. Improving rearing protocols, particularly with regard to food concentration and environmental conditions, is essential to completing the full development cycle.

Understanding the reproductive biology of *Pinna nobilis* in controlled environments will be critical to developing effective conservation strategies for this endangered species.

## 12. Conclusions

*Pinna nobilis*, the largest bivalve mollusc endemic to the Mediterranean, is now on the brink of collapse. This charismatic symbol of the Mediterranean is considered as a flagship species [121] of this large marine area and acts as a keystone architect of a vibrant underwater ecosystem, drawing public attention to the urgent need for the protection of fan mussels and other species within their habitat. Furthermore, as an umbrella species [122], it provides both direct and indirect refuge for countless marine organisms, and its protection has created a chain effect that strengthened the Mediterranean marine biodiversity over the past three decades.

In such a context, this review aims to provide a comprehensive overview of the conservation status of fan mussels, emphasising the paramount importance of protecting and preserving *Pinna nobilis* and its ecosystem. Conservation efforts for this bivalve must be intensified not only to prevent the loss of a single species but to safeguard the broader Mediterranean marine ecosystem, which represents a biodiversity heritage of incalculable value.

## Data Availability

No new data were created or analysed in this study. Data sharing is not applicable to this article.
